# The Discovery of New Drug-Target Interactions for Breast Cancer Treatment

**DOI:** 10.3390/molecules26247474

**Published:** 2021-12-10

**Authors:** Jiali Song, Zhenyi Xu, Lei Cao, Meng Wang, Yan Hou, Kang Li

**Affiliations:** 1 Department of Biostatistics, School of Public Health, Peking University, Beijing 100191, China; songjl@bjmu.edu.cn; 2 Department of Epidemiology and Biostatistics, School of Public Health, Harbin Medical University, Harbin 150086, China; xuzy@hrbmu.edu.cn (Z.X.); caolei@hrbmu.edu.cn (L.C.); wangm@hrbmu.edu.cn (M.W.)

**Keywords:** DTIs prediction, breast cancer, drug repurposing, machine learning, PsePSSM, DCCA coefficient

## Abstract

Drug–target interaction (DTIs) prediction plays a vital role in probing new targets for breast cancer research. Considering the multifaceted challenges associated with experimental methods identifying DTIs, the in silico prediction of such interactions merits exploration. In this study, we develop a feature-based method to infer unknown DTIs, called PsePDC-DTIs, which fuses information regarding protein sequences extracted by pseudo-position specific scoring matrix (PsePSSM), detrended cross-correlation analysis coefficient (DCCA coefficient), and an FP2 format molecular fingerprint descriptor of drug compounds. In addition, the synthetic minority oversampling technique (SMOTE) is employed for dealing with the imbalanced data after Lasso dimensionality reduction. Then, the processed feature vectors are put into a random forest classifier to perform DTIs predictions on four gold standard datasets, including nuclear receptors (NR), G-protein-coupled receptors (GPCR), ion channels (IC), and enzymes (E). Furthermore, we explore new targets for breast cancer treatment using its risk genes identified from large-scale genome-wide genetic studies using PsePDC-DTIs. Through five-fold cross-validation, the average values of accuracy in NR, GPCR, IC, and E datasets are 95.28%, 96.19%, 96.74%, and 98.22%, respectively. The PsePDC-DTIs model provides us with 10 potential DTIs for breast cancer treatment, among which erlotinib (DB00530) and FGFR2 (hsa2263), caffeine (DB00201) and KCNN4 (hsa3783), as well as afatinib (DB08916) and FGFR2 (hsa2263) are found with direct or inferred evidence. The PsePDC-DTIs model has achieved good prediction results, establishing the validity and superiority of the proposed method.

## 1. Introduction

Breast cancer is the most common gynecological malignant tumor in the world [1], with incidence rates that outdistance other cancers in both transitioned and transitioning countries [2]. It is reported that the global incidence of breast cancer has increased at a rate of 0.5% annually [3]. Actually, hereditary and genetic factors can account for 5% to 10% of breast cancer cases [2]. So far, approximately 100 breast cancer risk loci have been identified in a genome-wide association study (GWAS) [4]. However, only a few of targets are specifically for the development of new drugs for breast cancer. For example, in the ChEMBL dataset, there are 13 targets corresponding to 348 compounds, among which 209 compounds’ max phase is phase 4 in terms of breast cancer. Therefore, with the purpose of exploring new targets for drugs of breast cancer treatment, predicting new drug–target interactions (DTIs) is a good solution. The cost and time factors associated with the development of new drugs on a commercial scale [5,6,7] warrant the need for examining the already approved drugs. So, we explore new DTIs via drugs approved by FDA and the breast cancer risk genes identified from large-scale genome-wide genetic studies [8,9,10,11].

Wet lab experiments can infer the DTIs by using various techniques of classical and reverse pharmacology [12], while experimental methods identifying DTIs are expensive, time-consuming, and challenging. Therefore, for complementing experimental results, the in silico prediction of interactions between drugs and their targets is desirable [13]. The computational (in silico) methods for predicting drug–target interactions can be broadly classified into three categories: ligand-based approaches, docking-based approaches, and chemogenomic approaches [13].

For the first category, the main idea of ligand-based approaches is that similar molecules usually bind to similar protein targets and show similar properties [14,15]. However, the disadvantages of these approaches are that disregarding the information on the protein domain limits novel interactions to the link between known ligands and protein families, and hence, insufficient known ligands of target proteins may lead to poor performance [16]. As to the second category, docking approaches are powerful molecular modeling methods, which apply molecular dynamics using the 3D structures of the proteins as well as drugs to predict DTIs [17,18]. However, they cannot be applied in some cases where the 3D structures of proteins are not known. Most of the membrane proteins, for instance, have no three-dimensional structures in the freely protein databases. The third category, i.e., chemogenomic approaches, integrates the chemical information of the compounds and genomic information of proteins into a unified framework to predict DTIs. The preponderance of chemogenomic approaches is due to the fact that they overcome the disadvantages of ligand-based and docking-based approaches that have been discussed previously [19]. One of the chemogenomic methods categories, i.e., feature based methods, represents the drug-target pair with a vector of descriptors that may be produced by combining the properties of drug and targets, and can be put into various machine learning models to predict novel interactions [17].

In this study, we develop a feature-based method to infer unknown DTIs, called PsePDC-DTIs. The process of this method is described as follows. First, fusing protein features are generated by the pseudo-position specific scoring matrix (PsePSSM) algorithm, detrended cross-correlation analysis coefficient (DCCA coefficient), and FP2 fingerprint features of drug molecules under four benchmark datasets. Secondly, the least absolute shrinkage and selection operator (Lasso) method is used to reduce the dimension and noise information in the original high-dimensional space. Thirdly, the synthetic minority oversampling technique (SMOTE) is employed with Lasso feature-selected data for dealing with a high degree of imbalance in the samples used in this study. Finally, an ensemble classifier, random forest, is adopted to perform DTI predictions on four gold standard datasets, including nuclear receptors, G-protein-coupled receptors, ion channels, and enzymes. In the experiment, we make predictions concerning the gold standard DTI datasets by 5-fold cross-validation. Moreover, we can predict new DTIs for breast cancer with its risk genes by using PsePDC-DTIs.

## 2. Results

### 2.1. Performance Evaluation

In this study, the five-fold cross-validation approach is used to evaluate the performance of the prediction model. For each data set, all the DTIs are randomly divided into five parts of roughly equal size. Each part is taken in turn as the test set, while the remaining four parts serve as the training set to establish a prediction model.

The following parameters, Accuracy (ACC), Specificity (SP), and Sensitivity (SE), F score are calculated to assess the performance of the prediction model proposed in the experiment. The definition is as follows:

$$ACC=\frac{TP+TN}{TP+TN+FP+FN}$$

$$SE=\frac{TP}{TP+FN}$$

$$SP=\frac{TN}{TN+FP}$$

$$F=\frac{2TP}{2TP+FP+FN}$$

where true positive (TP) represents the number of positive pairs that are predicted to be interacting, whereas false positive (FP) is the count of negative pairs that are predicted to be interacting. Similarly, true negative (TN) is the total of negative pairs that are predicted to be non-interacting and false negative (FN) represents the number of positive pairs that are predicted to be non-interacting.

In addition, the receiver operating characteristic (ROC) is another important tool to assess the generalization performance of the model. ROC curve is a plot of the true positive rate (TPR) and false positive rate (FPR) which depicts the performance of a predictor at various threshold values. To compare these curves, area under the curve (AUC) is computed by summing the areas under the ROC curve.

A similar metric, the precision-recall curve (PR curve), can be obtained by using precision and recall at multiple threshold settings. The precision and recall ratio are defined as:

$$P=\frac{TP}{TP+FP}$$

$$R=\frac{TP}{TP+FN}$$

**Figure 1:**
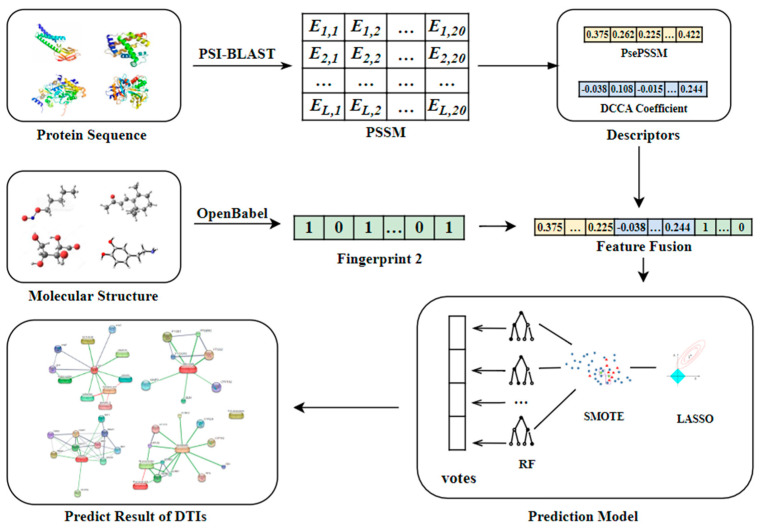
The workflow of PsePDC-DTIs.

Area under the precision-recall (AUPR) can also be obtained by summing the areas under the PR curve. For skewed datasets like the DTIs datasets in this paper, AUPR is of more significance because it penalizes the false positives more as compared to AUC, and is thus more suitable for imbalanced datasets. The higher the value of AUPR, the better [20,21]. The general framework of the PsePDC-DTIs model is shown in Figure 1 for an intuitive understanding.

### 2.2. Features Generation

#### 2.2.1. Parameter Setting for PsePSSM and DCCA Coefficient

Both the PsePSSM and the DCCA coefficient algorithms can control the validity of the algorithm to extract the feature information of the protein sequences by adjusting some of the parameters in the algorithm. The selection of parameters *λ* and *s* is very important for the accuracy of a protein-target interactions prediction model. In order to discover the merits of the feature parameters, we use the benchmark datasets as the research object, while the optimal values of *λ* and *s* are selected by the prediction accuracy and average prediction accuracy of the four datasets under different parameters [22,23].

In this paper, the *λ* value of PsePSSM algorithm indicates the sequence-order information of the amino acid residues in the protein sequence. To find the optimal *λ* value, we set the *λ* values from 0 to 15 in order. For the different *λ* values, the gold standard datasets enzymes, ion channels, GPCRs, and nuclear receptors are classified by RF and tested by 5-fold cross-validation respectively. The results can be seen in Supplementary Table S2. To find the best *λ* value more intuitively, the prediction accuracy and average accuracy with different *λ* values for the four datasets is shown in Supplementary Figure S1. In order to unify the parameters in the model, we take the *λ* value corresponding to the highest average accuracy for the optimal parameter which is up to 97.425% with *λ* = 3. Therefore, an 80-dimensional feature vector is acquired when using the PsePSSM method with the optimal parameter *λ* value of 3 to extract features of each target protein.

The *s* value of DCCA coefficient algorithm determines the length of each overlapping segment in which the covariance and variance of the residuals are calculated. In the gold standard datasets E, IC, GPCRs, and NR, the length of the shortest protein sequence is 83, therefore, the maximum *s* value is allowed for 82. To find the optimal *s* value, we set *s* values from 9 to 81 in turn. For the different *s* values, benchmark datasets are classified by RF and tested by 5-fold cross-validation respectively. In order to unify the parameters in the model, we take the *s* value corresponding to the highest average accuracy for the optimal parameter, which is up to 97.2925% when *s* = 36. The prediction accuracy and average prediction accuracy with different values of the four datasets is shown in Supplementary Figure S2 and Table S3.

#### 2.2.2. The Dimensionality of the Generated Features

We can obtain a 526-dimension feature vector which is composed of an 80-dimension vector generated by PsePSSM, 190-dimension vector generated by DCCA coefficient, and 256-dimension vector of the FP2 format molecular fingerprint.

### 2.3. Predictive Performance of Lasso for Dimensionality Reduction

As mentioned above, there are 526-dimension features for prediction, and the Lasso dimensionality reduction algorithm can extract useful information and discard redundancy from the complex information in the feature vector, which can improve the prediction process to some extent. The performance evaluation parameters for Lasso are shown in Supplementary Table S4. As we can see from Supplementary Table S4, the values of different indicators are comparable before and after using Lasso, which illuminates the ability of Lasso for extracting useful information.

### 2.4. Predictive Performance of SMOTE for Imbalanced Datasets

The classification of data with imbalanced class presents a significant drawback of the performance attainable using most standard classifier learning algorithms, which assume a relatively balanced class distribution and equal misclassification costs [24]. For this reason, as mentioned above, the SMOTE method has been used to convert the Lasso feature-selected data from imbalanced to balanced form, which is implemented in the DMwR R package where the oversampling parameter, the undersampling parameter, and the nearest neighbor algorithm parameter are set to 500, 120, and 5, respectively.

**Figure 2:**
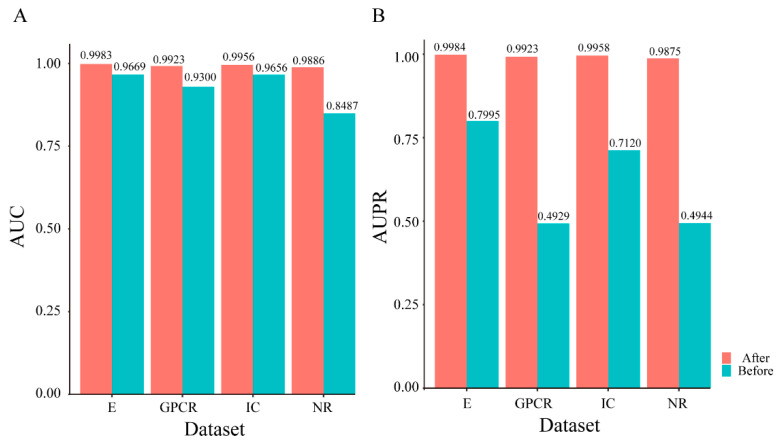
Predictive performance comparison of PsePDC-DTIs on benchmark datasets before and after SMOTE optimization in terms of AUC (**A**) and AUPR (**B**).

Due to the number of positive examples is much smaller than the number of negative examples, the indicators SE and SP are proportional to the correct proportion of positive and negative examples in the sample, and the indicator ACC has no significance in measuring the merits of the algorithm [23]. Therefore, the indicators that can reasonably measure the evaluation performance of the prediction model are AUC and AUPR among the above-mentioned indicators. To reflect the effect of data balance on the prediction performance of the model more directly, the visual display of the AUC and AUPR comparison under NR, GPCR, IC, and E datasets on unbalanced datasets and balanced datasets is shown in Figure 2. The evaluation indicators mentioned above are shown in Supplementary Table S5.

Figure 2 illuminates that the datasets after SMOTE processing have been vastly improved as far as AUC and AUPR are concerned. For the increase of AUC value after balancing, the highest is in the NR dataset with 0.1399, followed by GPCR dataset with 0.0623, E dataset with 0.0314, and IC dataset with 0.0300. As to AUPR, the highest is in GPCR dataset with 0.4994, followed by NR dataset with 0.4931, IC dataset with 0.2838, and E dataset with 0.1989. So, we can conclude that SMOTE processing lead to a greater improvement in the prediction performance.

### 2.5. Predictive Performance of RF for DTIs Prediction

A classifier plays an important role in the quality of a prediction model, and thus might influence the prediction performance. In order to explore the machine learning (ML) methods which are used frequently, we investigate seven common classification algorithms of ML (i.e., random forest, naïve Bayes, decision tree, support vector machine, oneR, k-nearest neighbors, repeated incremental pruning to produce error reduction).

To ensure fairness, the target protein sequences are extracted by PsePSSM and DCCA coefficient for the four datasets constructed, and the drug compounds are expressed by FP2 format molecular fingerprint descriptor. After fusing the features, the Lasso method for dimensionality reduction and SMOTE method for skewed datasets are used. To obtain robust results and accurate comparison, we keep the same experimental conditions, where the same training drugs-target interaction pairs and test drugs-target interaction pairs are used across the seven classifiers in each cross-validation [25]. The prediction results on four datasets of seven classifiers are shown in Supplementary Table S6.

From the boldfaced fonts in Supplementary Table S6, we observe that RF significantly outperformed the other machine learning methods under four datasets in terms of ACC, SP, F, AUC, and AUPR metrics. However, for the SE metric, SVM secured the first position with SE values of 96.98%, 97.50%, and 97.81% which is 1.15%, 1.96%, and 0.20% higher than RF in GPCR, IC, and E datasets, respectively. However, each of the SE values in these three datasets is over 95%, which means that more than 95% of actual DTIs can be correctly identified.

**Figure 3:**
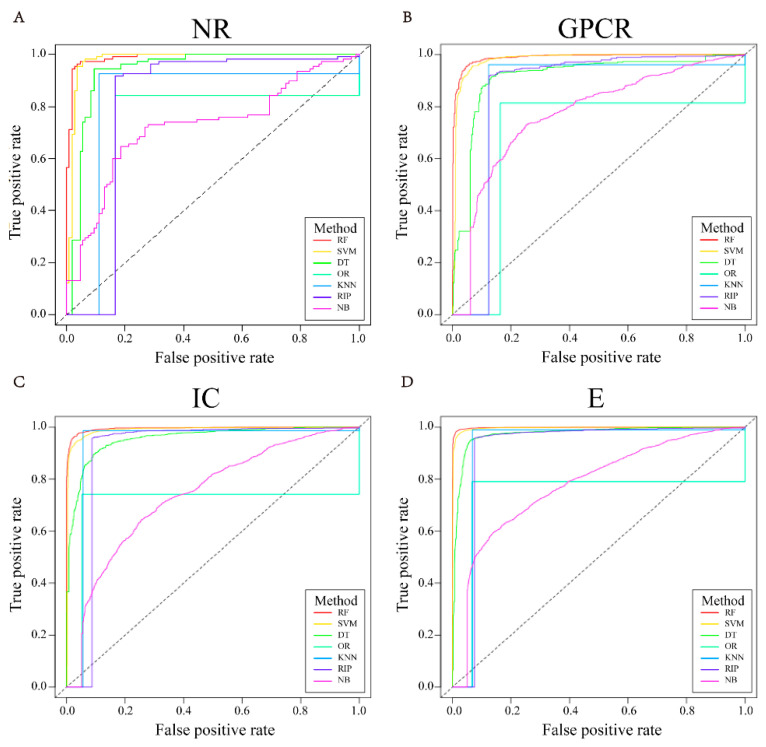
The representative ROC curves of different classifiers in 5-fold cross-validation of four datasets, i.e., NR (**A**), GPCR (**B**), IC (**C**), E (**D**).

**Figure 4:**
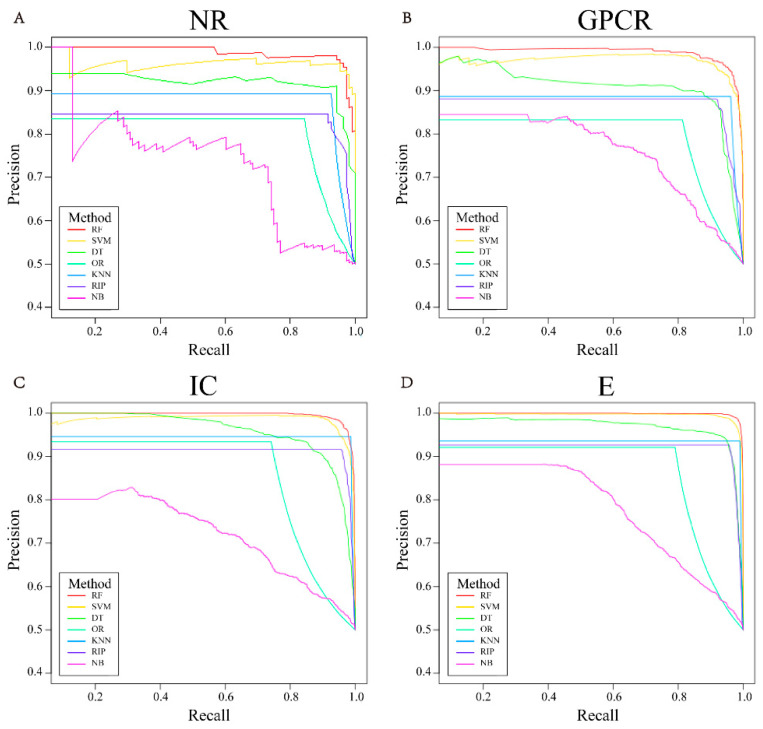
The representative PR curves of different classifiers in 5-fold cross-validation of four datasets, i.e., NR (**A**), GPCR (**B**), IC (**C**), E (**D**).

Figure 3 shows one of the ROC curves of seven different classification algorithms under the NR, GPCR, IC, and E datasets in five-fold cross-validation, while other ROC curves are shown in Supplementary Figure S3. Figure 4 reveals one of the PR curves of seven different classifiers under four datasets in five-fold cross-validation, while other PR curves can be found in Supplementary Figure S4.

According to Figure 3 and Figure 4, the ROC and PR curves of the four datasets almost surround others with random forest as the classifier, and the corresponding AUC and AUPR values are also larger. Therefore, we choose random forest as the classification algorithm of the prediction model.

### 2.6. Predictive Performance of PsePDC-DTIs Compared with State-of-the-Art Methods

There are a variety of prediction models proposed for detecting DTIs. Our method applies LASSO to select features and SMOTE to balance data for DTIs under gold standard datasets and evaluates prediction performance based on five-fold cross-validation. To further expound the efficiency of the predictor in this study, we compared our prediction performance with other methods which also used the same benchmark datasets and tested by five-fold cross-validation [16]. Table 1 lists the comparison results of other models, including NetCBP [26], Huang et al. [27], Bigram-PSSM [21], iDTI-ESBoost [20], Li et al. [28], KBMF2K [29], and NRLMF [30]. It can be seen that our predictor PsePDC-DTIs achieves AUC values of 0.9886, 0.9923, 0.9956, and 0.9983 on the NR, GPCR, IC, and E datasets, respectively, which significantly outperforms other methods for all datasets.

Moreover, Mousavian et al. [21] argued that the AUPR is a more accurate measure for evaluating performance in dealing with highly imbalanced datasets compared to the AUC for the reason that the highly ranked false positive samples are punished by the AUPR much more than the AUC. To compare the performance in terms of AUPR among Bigram-PSSM [21], iDTI-ESBoost [20], and NRLMF [30], we reported the AUPR values of the three predictors in Table 2. The AUPR values of our model PsePDC-DTIs are 0.9875, 0.9923, 0.9958, and 0.9984 on the NR, GPCR, IC, and E datasets, respectively. This clearly shows that our method PsePDC-DTIs outperforms other methods in terms of AUPR as well.

The values of AUC and AUPR demonstrated above indicate the effectiveness of the extracted feature information, dimensionality reduction of features, balancing methods, and classifier proposed in this research.

### 2.7. Predictive Performance of PsePDC-DTIs Compared with State-of-the-Art Methods

According to the information introduced above, we can confirm the reliability of our proposed model. In the inference process, we use all the known drugs and target proteins in the gold standard datasets as training data, and predict potential interactions between 52 human proteins and 1556 FDA approved drugs as mentioned in the datasets section.

**Figure 5:**
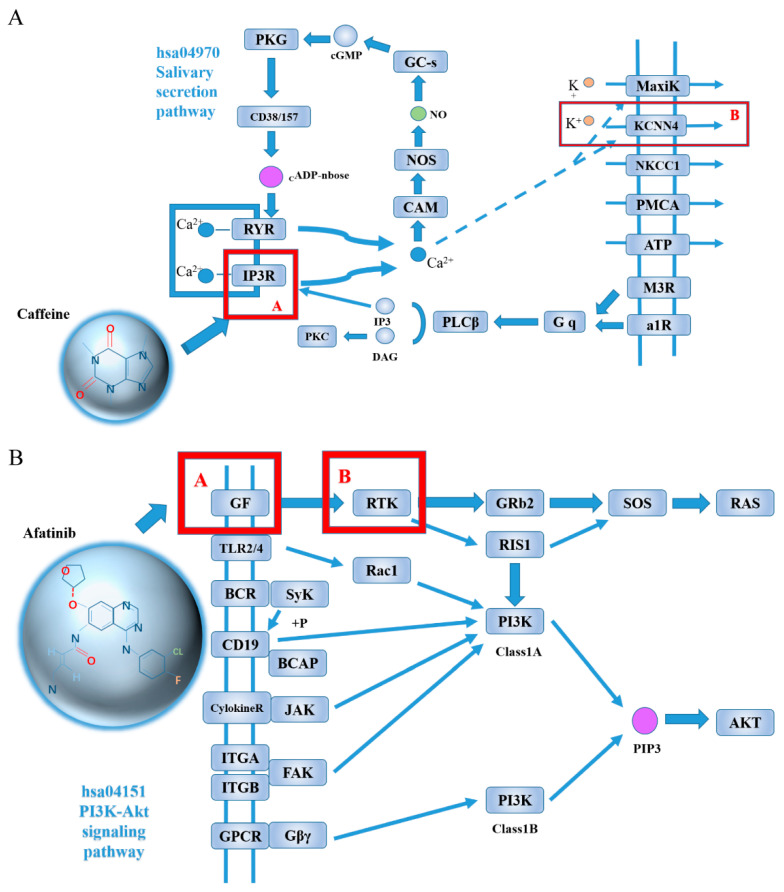
The potential mechanism illustration of predicted DTIs of Caffeine and KCNN4 (**A**), Afatinib and FGFR2 (**B**). In Figure 5B, GF contains EGF, RTK contains targets of EGFR, ERBB2, ERBB4, FGFR2.

For the 52 breast cancer target proteins and the PsePDC-DTIs model trained in gold standard datasets, we predict all the DTIs mentioned in Section 4.1.2 and rank them by their probability. There are 383 predicted DTIs with a probability greater than 0.5 reported in Supplementary Table S7, which means 0.47% pairs were predicted as interaction. This is in line with the fact that the number of non-interacting pairs is far more than the number of interaction pairs [21]. We extract the top 10 drug–target pairs ranked by their prediction probability values, as listed in Table 3, and present the potential mechanism of predicted DTIs in Figure 5. Figure 5A shows that IP3R, the target of caffeine (DB00201), regulates KCNN4 via Ca^2+^ in the salivary secretion pathway. Figure 5B demonstrates that GF, the target of afatinib (DB08916), regulates RTK directly.

## 3. Discussion

As shown above, the average values of accuracy in GPCR, IC, and E datasets are 95.28%, 96.19%, 96.74%, and 98.22%, respectively. The average AUC achieves 0.9886, 0.9923, 0.9956, and 0.9983 on the NR, GPCR, IC, and E datasets, respectively, which outperformed some methods reported elsewhere [20,21,26,27,28,29]. In the literature [21], it has been revealed that AUPR is the most appropriate metric for the comparison of such imbalanced datasets. Moreover, the average AUPR of PsePDC-DTIs outperformed other methods [20,21], reaching 0.9875, 0.9923, 0.9958, and 0.9984 under these four datasets, respectively.

During predicting DTIs between 52 human proteins of breast cancer and 1556 FDA approved drugs in the DrugBank database, our comprehensive model provides us with 10 potential DTIs, among which three DTIs are found with direct or inferred evidence. There is direct evidence about the DTIs of erlotinhas(DB00530) and FGFR2 (hsa2263) in SuperTarget. In addition, we obtain indirect evidence of predicted DTIs when the known target for a drug regulates the predicted target for this drug by using pathways from the KEGG database. Figure 5A shows that the target IP3R for caffeine (DB00201), which can be found in DrugBank datasets, regulates KCNN4 via Ca^2+^ in the salivary secretion pathway. This is important with respect to the fact that several studies demonstrate the relationship of salivary to breast cancer [31,32,33,34,35,36,37,38]. For example, Sawczuk et al. [36] indicated that salivary peroxidase may have particular clinical significance in non-invasive diagnostics of breast cancer. Liu et al.’s study [37] contributed to the screening of patients with early-stage breast cancer based on precise alterations of salivary glycopatterns.

Furthermore, we find six pathways to explain the relationship between afahasib (DB08916) and FGFR2 (hsa2263). Taking PI3K-Akt signaling pathway as an example, Figure 5B shows that the target GF (contains EGF) for afatinib (DB08916) which can be found in DrugBank datasets regulates RTK (contains targets of EGFR, ERBB2, ERBB4, FGFR2) directly. Again, this is significant as several studies suggest that PI3K-Akt signaling pathway is connected with breast cancer [39,40,41,42,43,44,45,46,47,48,49,50,51,52,53,54,55,56,57,58,59,60,61]. Chandarlapaty et al. [44] prospectively collected trastuzumab-refractory human breast cancers, and found that activation of the PI3K-Akt pathway through loss of PTEN or PIK3CA mutation was frequently observed. Other pathways about afatinib (DB08916) and FGFR2 (hsa2263) can be found in Supplementary Figure S5.

In the remaining predicted seven DTIs, although we could not find any evidence from databases, pathways, and literature, they still have the potentiality to be true positive DTIs [62]. For instance, some researches [63] propose that theophylline (DB00277) and caffeine (DB00201) are often regarded as a group which is related to breast cancer. Thus, it is possible that both drugs interact with the same target.

However, if the drug–target interactions dataset as training data is too large, the PsePDC-DTIs model cannot predict drug–target interactions rapidly because we use RF as classifier. Therefore, in order to improve the operating speed of the proposed model and keep the prediction accuracy, in the future, we will attempt to use a deep learning network as classifier. Moreover, to handle the class imbalance problem, our proposed model used SMOTE to generate artificial examples for the minority class. However, during the cross-validation process, the test dataset also contains the artificial examples generated by SMOTE, which may cause the current reported prediction performance exaggeration. Therefore, we will explore a more conservative and effective method for dealing with imbalanced data. In addition, further research into the new methods of the features will be essential because the algorithm of extracting the feature information of the protein sequences and drug compounds is very important for the performance of a protein–target interactions prediction model.

## 4. Materials and Methods

### 4.1. Datasets

#### 4.1.1. Benchmark Datasets

The benchmark datasets are used for assessing the performance of PsePDC-DTIs by five-fold cross-validation. For this study, they are the gold standard datasets studied by Yamanishi et al. [64], obtained from http://web.kuicr.kyoto-u.ac.jp/supp/yoshi/drugtarget/ (accessed on 9 December 2021). All data concerning DTIs pairs in the gold standard datasets are collected from the KEGG BRITE [65], BRENDA [66], SuperTarget [67], and Drug Bank databases [68]. The drug target links have been considered for four protein targets, namely enzymes(E), ion channels (IC), G-protein-coupled receptors (GPCR), and nuclear receptor (NR). As listed in Table 4, the number of known drugs target in these classes is 445, 210, 223, and 54, respectively, and the number of proteins known to be targeted by the drugs in these classes is 664, 204, 95, and 26 respectively. Among these drug-target pairs, 5127 pairs are known to interact with each other, and the number of interacting pairs in each class is 2926, 1476, 635, and 90 respectively.

For a completely connected bipartite graph, there must be drugs × targets connections. Taking the enzyme dataset, for instance, there exist 455 × 664 = 302,120 drug-target pairs. In our study, 5172 pairs which are known to interact with each other are used as the positive samples while the rest of connections are considered negative samples. The number of samples for four datasets is listed in Table 4.

#### 4.1.2. DTIs Dataset Constructed by Drugs of FDA-Approved and Targets of Breast Cancer

In order to predict new DTIs of breast cancer treatment for drugs approved by the FDA, we propose a DTIs dataset whose drugs are from a dataset named DrugBank_approved [69] which contains 1556 FDA-approved drugs until 2016. As to the targets, we use the 110 putative target genes of breast cancer identified by Baxter et al. [10] and 179 genes whose predicted expression was associated with breast cancer risk [11] for drug repurposing. According to these 286 genes (removing 3 duplicates), we obtain 52 human proteins annotated as members of the four classes of target proteins (NR, GPCR, IC, and E) in KEGG GENES, which are listed in Supplementary Table S1. The DTIs which are generated by connecting each target protein with each drug molecule (only target protein and drug can be linked by aside) can be used for drug repurposing of breast cancer.

### 4.2. Methods for Features Generation

#### 4.2.1. Pseudo-Position Specific Scoring Matrix (PsePSSM)

The PsePSSM algorithm employed in the study is proposed by K.C. Chou [70]. PsePSSM is the extraction of the features of protein sequences, which can be obtained by translating the position specific scoring matrix (PSSM) of different dimensions for different protein sequences into the same dimension. The uniform vector is convenient for our subsequent study. PSSM [71] represents the evolutionary information of the protein sequences, which needs to blast the protein FASTA file against the UniProt database for constructing through PSI-BLAST [72]. For this study, the parameters of PSI-BLAST are set with three iterations, E-value is equal to 0.001, while the rest of the parameters are set by default. The constructed PSSM format for a protein sequence P with L amino acid residues is shown as formula (1). The rows of PSSM inform the corresponding amino acid positions in the protein sequence P, and columns of PSSM indicate the 20 native amino acid types that may be mutated.

(1) $$P_{PSSM}=\left[\begin{array}{cccccc} E_{1,1} & E_{1,2} & \cdots & E_{1,j} & \cdots & E_{1,20}\\ E_{2,1} & E_{2,2} & \cdots & E_{2,j} & \cdots & E_{2,20}\\ \vdots & \vdots & \cdots & \vdots & \cdots & \vdots \\ E_{i,1} & E_{i,2} & \cdots & E_{i,j} & \cdots & E_{i,20}\\ \vdots & \vdots & \cdots & \vdots & \cdots & \vdots \\ E_{L,1} & E_{L,2} & \cdots & E_{L,j} & \cdots & E_{L,20} \end{array}\right]$$

where $E_{i,j}$ represents the value of the residue of the *i*-th position in the amino acid sequence being mutated to the *j*-th native amino acid residue.

The elements of PSSM are normalized by formula (2), whose PSSM value ranges from 0 to 1, while the value in the original PSSM matrix ranges from −9 to 11.

(2) $$E_{i,j}^{\prime }=\frac{1}{1+e\left(-E_{i,j}\right)}$$

Because proteins with different lengths will correspond to matrices with different numbers of rows, in order to make the PSSM descriptor a uniform representation, a protein sequence P is represented by formula (3):

(3) $${\bar{P}}_{PSSM}={\left[\begin{array}{cccccc} {\bar{E}}_{1} & {\bar{E}}_{2} & \cdots & {\bar{E}}_{j} & \cdots & {\bar{E}}_{20} \end{array}\right]}^{T}$$

where ${\bar{E}}_{j}=\frac{1}{L}\overset{L}{\underset{i=1}{\sum }}E_{i,j}^{\prime }$, ${\bar{E}}_{j}$ manifests the average score of the amino acid residue in protein *P* being mutated to *j* amino acid type during the process of evolution.

Next, we transform PSSM of a single protein into a feature vector PsePSSM, as formula (4) shown.

(4) $$P_{PsePSSM}={\left[\begin{array}{cccccccccc} {\bar{P}}_{PSSM}{}^{T} & \theta_{1}^{1} & \theta_{2}^{1} & \cdots & \theta_{20}^{1} & \cdots & \theta_{1}^{\lambda } & \theta_{2}^{\lambda } & \cdots & \theta_{20}^{\lambda } \end{array}\right]}^{T}$$

where $\theta_{j}^{\lambda }=\frac{1}{L-\lambda }\overset{L-\lambda }{\underset{i=1}{\sum }}{\left(E_{i,j}^{\prime }-E_{(i+\lambda ),j}^{\prime }\right)}^{2}(\lambda <L;j=1,2,\cdots ,20)$, $\theta_{j}^{\lambda }$ is the correlation factor by coupling the *λ*-th-most contiguous PSSM scores along the protein chain for the amino acid type *j*. Therefore, a protein sequence generates a 20 + 20 × *λ*-dimensional feature vector using PsePSSM algorithm. PsePSSM matrix can be regarded as PSSM matrix when *λ* = 0. For this study, the optimal parameter of *λ* needs to be selected, so that the highest accuracy of a protein–target interactions prediction model is obtained.

#### 4.2.2. Detrended Cross-Correlation Analysis Coefficient (DCCA Coefficient)

Using the detrended cross-correlation analysis coefficient method, more protein information that truly reflects protein samples’ intrinsic correlation could be extracted from the PSSM matrix. DCCA coefficient was initially proposed by Podobnik and Stanley [73], which can be used to quantify the level of cross-correlation between two non-stationary time series [74]. Here, each amino acid is taken as one property and the PSSM including the evolutionary information expression is considered as the time series of all properties. The 20 columns in the PSSM matrix are considered to be 20 non-stationary time series [22,75].

For two arbitrary different columns of a normalized PSSM, $\left\{x_{i}\right\}$ and $\left\{y_{i}\right\}$ (i=1,2,⋯,L), new time series $X_{k}$ and $Y_{k}$ are calculated by using formula (5).

(5) $$\{\begin{array}{c} X_{k}=\begin{array}{cc} \overset{k}{\underset{i=1}{\sum }}x_{i} & k=1,2,\cdots ,L \end{array}\\ Y_{k}=\begin{array}{cc} \overset{k}{\underset{i=1}{\sum }}y_{i} & k=1,2,\cdots ,L \end{array} \end{array}$$

Then, the integrated time series $X_{k}$ and $Y_{k}$ are divided into (*L* − *s*) overlapping segments, and each segment which starts at *i* and ends at *I* + *s* contains (*s* + 1) values. For each segment of the data, the fitting values ${\tilde{X}}_{i,k}$ and ${\tilde{Y}}_{i,k}\left(i\leq k\leq i+s\right)$ can be obtained by the least squares linearly fitting. The covariance and variance of the residuals in each segment are calculated by formula (6)–(8):

(6) $$f_{xy}^{2}\left(s,i\right)=\frac{1}{s+1}\sum_{k=i}^{i+s}(X_{k}-{\tilde{X}}_{i,k})\left(Y_{k}-{\tilde{Y}}_{i,k}\right)$$

(7) $$f_{xx}^{2}\left(s,i\right)=\frac{1}{s+1}\sum_{k=i}^{i+s}{\left(X_{k}-{\tilde{X}}_{i,k}\right)}^{2}$$

(8) $$f_{yy}^{2}\left(s,i\right)=\frac{1}{s+1}\sum_{k=i}^{i+s}{\left(Y_{k}-{\tilde{Y}}_{i,k}\right)}^{2}$$

Next, we average all (*L* − *s*) overlapping segments and obtain the fluctuation function shown in formula (9)–(11):

(9) $$f_{xy}^{2}\left(s\right)=\frac{1}{L-s}\sum_{i=1}^{L-s}f_{xy}^{2}\left(s,i\right)$$

(10) $$f_{xx}^{2}\left(s\right)=\frac{1}{L-s}\sum_{i=1}^{L-s}f_{xx}^{2}\left(s,i\right)$$

(11) $$f_{yy}^{2}\left(s\right)=\frac{1}{L-s}\sum_{i=1}^{L-s}f_{yy}^{2}\left(s,i\right)$$

Finally, the DCCA coefficient of two different time series $\left\{x_{i}\right\}$ and $\left\{y_{i}\right\}$ is defined as formula (12). Hence, for the 20 columns in the PSSM matrix considered to be 20 non-stationary time series, a 190-dimensional feature vector will be generated for a certain *s* via the DCCA coefficient algorithm. We need to select the optimal parameter of *s* to obtain the highest accuracy of a protein–target interactions prediction model.

(12) $$\rho_{DCCA}\left(s\right)=\frac{f_{xy}^{2}\left(s\right)}{f_{xx}\left(s\right)f_{yy}\left(s\right)}$$

The value of $\rho_{DCCA}$ ranges from $-1\leq \rho_{DCCA}\leq 1$. Logically, 1 means perfect cross-correlation, 0 represents no cross-correlation, and −1 indicates perfect anti-cross-correlation [76].

#### 4.2.3. FP2 Molecular Fingerprint

Drug compounds are expressed by FP2 format molecular fingerprint descriptor that can be converted to a decimal digit sequence between 0 and 15 as a drug molecule 256-dimensional vector using OpenBabel Software (available from http://openbabel.org, accessed on 9 December 2021) [23].

### 4.3. Lasso for Dimensionality Reduction of Features

Shi et.al [23] proved that the least absolute shrinkage and selection operator (Lasso) method can effectively reduce information redundancy and delete some unimportant features compared with principal components analysis (PCA), ReliefF, and Elastic net. Therefore, we use Lasso as the dimensionality reduction algorithm for this paper. LASSO proposed by Tibshirani [77] is a compression estimation method with $l_{1}$ regularization implemented to achieve a sparse solution. LASSO is used to perform feature selection by forcing many parameters corresponding to the irrelevant and redundant features to zero value, and retaining the features corresponding to the non-zero coefficients for subsequent classification [78,79,80]. The aim of this approach is to minimize the cost function:

(13) $$\sum_{n=1}^{N}{\left(y_{n}-\sum_{m}x_{nm}\beta_{m}\right)}^{2}+\lambda \sum_{m=1}^{M}\left|\beta_{m}\right|$$

where $y_{n}$ represents the corresponding response vector of a DTI pair, that is, the class label of the sample, *N* is the number of samples, $x_{nm}$ is the *m*-th feature of the *n*-th sample, *λ* is the regularization parameter, and $\beta_{m}$ is the regression coefficients of *m*-th feature [78].

Therefore, through formula (13), we eliminate the noise and redundant information contained in the high-dimensional data obtained after the original drug and target feature extraction

### 4.4. SMOTE for High-Dimensional Class-Imbalanced Data

As shown in Table 4, there are severe imbalance problems between the positive and negative samples of four gold standard datasets. The ratio of negative samples to positive samples (sample ratio) is used for measuring the degree of imbalance. There is a high degree of imbalance in the enzyme dataset with the sample ratio reaching 99.98. In contrast, the nuclear receptor dataset has a low degree of imbalance, with a sample ratio that barely reaches 14.60. In order to deal with imbalanced data, some important techniques are proposed, such as random undersampling, random oversampling, and the synthetic minority oversampling technique (SMOTE). SMOTE overcomes imbalances by generating artificial data, while random undersampling and random oversampling replicate and add the observations from the minority class [80]. Therefore, this study uses SMOTE, which is a powerful method and creates artificial data based on feature space similarities from minority samples to handle the problems.

SMOTE, proposed by Chawla et al. [81], is one of the most popular oversampling methods. Its main idea is to interpolate a new synthetic minority class sample on the line that connects a randomly chosen minority class sample and one of its k-nearest neighbors belonging to the minority class samples. Specifically speaking, for each positive sample *z*, one gets its k-nearest neighbors from other positive samples. Then, one chooses one positive sample $\bar{z}$ among the neighbors [82]. Finally, this generates the synthetic sample $z_{new}$ by inserting between z and $\bar{z}$ as follows:

(14) $$z_{new}=z+rand\left(0,1\right)\times \left(\bar{z}-z\right)$$

where rand(0,1) refers to generate a random number between 0 and 1. Thus, a new, more balanced dataset is formed.

### 4.5. RF for DTIs Prediction

Random forest (RF) [83] is one of the famous bagging techniques based on decision tree models which is fast, robust to noise, does not overfit, but provides possibilities for the explanation and visualization of its output. In this study, RF was applied as a classification method by constructing a multitude of decision trees at training time and outputting the number of votes cast of all the trees [84]. Supposing the number of training cases were *P* and the total number of features in the classifier were *Q*. After making *p* bootstrap sample sets from the original training sample set, set up an unpruned tree with each sample set. At each node of the tree, randomly choose *q* features (*q* < *Q*) as a candidate variable on which to make the decision at that node [85]. With the generation of multiple classification trees, a random forest is built

## 5. Conclusions

In this paper, we develop a novel method for predicting and identifying DTIs, called PsePDC-DTIs. Specifically, the proposed method combines the pseudo-position specific scoring matrix (PsePSSM) and detrended cross-correlation analysis coefficient (DCCA coefficient) to extract the features of the protein sequences, for which PsePSSM feature extraction considers the sequence-order information of the protein sequence, and the DCCA coefficient uses the columns in the PSSM as the least squares fitting and the trend elimination as the non-stationary time series to remove the PSSM between the cross-correlation [22]. When using PsePSSM and DCCA coefficient, *λ* = 3 and *s* = 36 are selected, respectively. Drug compounds are expressed by FP2 format molecular fingerprint descriptor. The redundant information in the drug–target datasets is effectively removed by least absolute shrinkage and selection operator (Lasso). For dealing with the high degree of imbalance in the samples used in this study, the synthetic minority oversampling technique (SMOTE) is employed. The classification algorithm to predict DTIs is the random forest (RF) classifier. The five-fold cross-validation method is used in this work to assess the predictive performance of PsePDC-DTIs on four benchmark datasets. The PsePDC-DTIs model has achieved good prediction results, which shows that the proposed method is better than the state-of-art methods and appropriately designed.

## Figures and Tables

**Table 1:** Performance comparison of different approaches on benchmark datasets in terms of AUC.

Models	NR	GPCR	IC	E
NetCBP [26]	0.8394	0.8235	0.8034	0.8251
Huang et al. [27]	0.9634	0.9053	0.9382	0.9601
Bigram-PSSM [21]	0.8690	0.8720	0.8890	0.9480
iDTI-ESBoost [20]	0.9285	0.9322	0.9369	0.9689
Li et al. [28]	0.9300	0.9171	0.8856	0.9288
KBMF2K [29]	0.8240	0.8570	0.7990	0.8320
NRLMF [30]	0.9500	0.9690	0.9890	0.9870
**PsePDC-DTIs**	**0.9886**	**0.9923**	**0.9956**	**0.9983**

**Table 2:** Performance comparison of different approaches on benchmark datasets in terms of AUPR.

Models	NR	GPCR	IC	E
Bigram-PSSM [21]	0.4110	0.2820	0.3900	0.5460
iDTI-ESBoost [20]	0.7900	0.5000	0.4800	0.6800
NRLMF [30]	0.7280	0.7490	0.9060	0.8920
**PsePDC-DTIs**	**0.9875**	**0.9923**	**0.9958**	**0.9984**

**Table 3:** Drug-target pairs ranked by prediction probability.

Drug	Drug_Name	Target	Target_Name	Prob
**DB00201**	**Caffeine**	**hsa3783**	**KCNN4**	**0.988**
DB00277	Theophylline	hsa3783	KCNN4	0.982
DB01412	Theobromine	hsa3783	KCNN4	0.93
DB00530	Erlotinib	hsa238	ALK	0.886
DB00806	Pentoxifylline	hsa3783	KCNN4	0.884
DB00824	Enprofylline	hsa3783	KCNN4	0.866
**DB00530**	**Erlotinib**	**hsa2263**	**FGFR2**	**0.864**
DB00661	Verapamil	hsa57719	ANO8	0.846
DB01303	Oxtriphylline	hsa3783	KCNN4	0.844
**DB08916**	**Afatinib**	**hsa2263**	**FGFR2**	**0.806**

**Table 4:** The benchmark data sets used in this study.

Datasets	Drugs	Targets	Interactions	Positive Samples	Negative Samples	Sample Ratio
Enzyme	445	664	2926	2926	292,554	99.98
IC	210	204	1476	1476	41,364	28.02
GPCR	223	95	635	635	20,550	32.36
NR	54	26	90	90	1314	14.60
Total	932	989	5127	5127	355,782	-

## Data Availability

The datasets generated during and analysed during the current study are available in the github repository, https://github.com/SongJialiJiali/PsePDC-DTIs-model.git (accessed on 9 December 2021).

## Supplementary Materials

The following are available online, Figure S1: Prediction result of selecting different *λ* on four datasets, Figure S2: Prediction result of selecting different *s* on four datasets, Figure S3: the ROC curves of different classifiers in 5-fold cross-validation, Figure S4: the PR curves of different classifiers in 5-fold cross-validation, Figure S5: the inferred evidence for DTIs of afatinib (DB08916) and FGFR2 (hsa2263). (a) RTK contains EGFR, FGFR2, GF contains EGF; (b) GF contains EGF, RTK contains FGFR2, EGFR; (c) GF contains EGF, GFR contains EGFR, ERBB2, FGFR2; (d) RTK contains EGFR, ERBB2, ERBB4, FGFR2, GF contains EGF; (e) RTK contains FGFR2, EGFR, GF contains EGF. Table S1: Targets for drug repurposing of breast cancer, Table S2: Prediction result of selecting different *λ* on four datasets, Table S3: Prediction result of selecting different *s* on four datasets, Table S4: Prediction results on four datasets before and after Lasso for dimensionality reduction, Table S5: Prediction results on four datasets before and after SMOTE optimization, Table S6: Prediction results on four datasets of seven classifiers, Table S7: Drug-target interaction pairs with a probability score no less than 0.5.
