# Supplementary material for: The Discovery of New Drug-Target Interactions for Breast Cancer Treatment
Source: Molecules. 2021 Dec 10;26(24):7474. doi: 10.3390/molecules26247474 (PMC8704452; doi:10.3390/molecules26247474)
Supplement: Supplementary file 1 [file molecules-26-07474-s001.zip › molecules-1497256-supplementary.pdf]

Supplementary materials:

The discovery of new drug-target interactions for breast cancer treatment

Jiali Song [1,+], Zhenyi Xu [2,+], Lei Cao [2], Meng Wang [2], Kang Li [2,*] and Yan Hou [1,*]

1 Department of Biostatistics, Peking University, Beijing 100000, China; songjl@bjmu.edu.cn; houyan@bjmu.edu.cn
2 Department of Epidemiology and Biostatistics, School of Public Health, Harbin Medical University, Harbin, 150086, P.R. China; xuzy@hrbmu.edu.cn; caolei@hrbmu.edu.cn; wangm@hrbmu.edu.cn; likang@ems.hrbmu.edu.cn
* Correspondence: YH: houyan@bjmu.edu.cn; Tel.: +86-010-8280-5952 and KL: likang@ems.hrbmu.edu.cn; Tel.: +86-0451-8750-2679
\+ these authors contributed equally to this work

Table S1 Targets for drug repurposing of breast cancer.

Gene	Target	Dataset
CTSW	hsa1521	Enzymes
FGFR2	hsa2263	Enzymes
GTPBP3	hsa84705	Enzymes
HIPK1	hsa204851	Enzymes
IGHMBP2	hsa3508	Enzymes
ISYNA1	hsa51477	Enzymes
KAT5	hsa10524	Enzymes
MAP3K1	hsa4214	Enzymes
MTAP	hsa4507	Enzymes
MUS81	hsa80198	Enzymes
NGLY1	hsa55768	Enzymes
OXSM	hsa54995	Enzymes
PGD	hsa5226	Enzymes
PGD	hsa26227	Enzymes
PGPEP1	hsa54858	Enzymes
PPA2	hsa27068	Enzymes
RAD18	hsa56852	Enzymes
USE1	hsa65264	Enzymes
MRGPRF	hsa116535	G protein-coupled receptors
ANO8	hsa57719	ion channels
KCNN4	hsa3783	ion channels
TPCN2	hsa219931	ion channels
HNF4G	hsa3174	Nuclear receptors
NR2F6	hsa2063	Nuclear receptors
GALNT16	hsa64409	Enzymes
CASP8	hsa841	Enzymes
UBLCP1	hsa134510	Enzymes

Gene	Target	Dataset
B3GNT1	hsa10678	Enzymes
MAN2C1	hsa4123	Enzymes
CRHR1	hsa1394	G protein-coupled receptors
ALK	hsa238	Enzymes
FES	hsa2242	Enzymes
GSTM4	hsa2948	Enzymes
APOBEC3A	hsa100913187	Enzymes
GSTM3	hsa2947	Enzymes
CCBL2	hsa56267	Enzymes
CMTR2	hsa55783	Enzymes
MTHFD1L	hsa25902	Enzymes
METTL10	hsa399818	Enzymes
GPR144	hsa347088	G protein-coupled receptors
DGKQ	hsa1609	Enzymes
MUTYH	hsa4595	Enzymes
TRIM4	hsa89122	Enzymes
PTDSS2	hsa81490	Enzymes
GSTM5	hsa2949	Enzymes
KDM6B	hsa23135	Enzymes
C9orf3	hsa84909	Enzymes
GPR156	hsa165829	G protein-coupled receptors
BDH2	hsa56898	Enzymes
USP19	hsa10869	Enzymes
MMP24	hsa10893	Enzymes
NR1H3	hsa10062	Nuclear receptors

Table S2 Prediction result of selecting different λ on four datasets.

λ	5-fold cross-validation (%)															
	**0**	**1**	**2**	**3**	**4**	**5**	**6**	**7**	**8**	**9**	**10**	**11**	**12**	**13**	**14**	**15**
NR	94.52	94.8	94.73	**94.94**	94.8	94.66	94.8	94.73	94.66	94.66	94.73	94.8	94.59	94.8	94.73	94.8
GPCR	97.31	**97.46**	97.45	**97.46**	**97.46**	97.45	**97.46**	97.44	97.41	97.42	97.42	97.43	97.43	97.43	97.43	97.44
IC	97.27	97.76	**97.78**	97.76	97.74	97.71	97.69	97.68	97.67	97.67	97.67	97.64	97.64	97.63	97.61	97.61
E	99.35	99.51	99.54	99.54	**99.55**	**99.55**	99.54	99.54	99.54	99.54	**99.55**	**99.55**	99.54	99.54	99.54	**99.55**
Average	97.1125	97.3825	97.375	**97.425**	97.3875	97.3425	97.3725	97.3475	97.32	97.3225	97.3425	97.355	97.3	97.35	97.3275	97.35

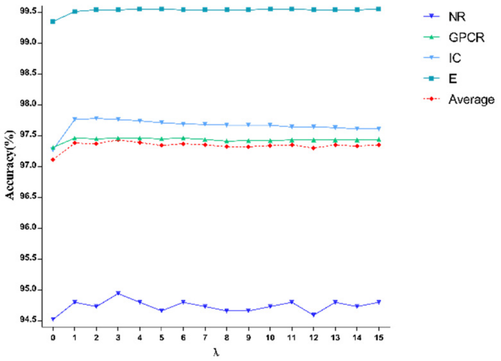

Figure S1 Prediction result of selecting different *λ* on four datasets

Table S3 Prediction result of selecting different *s* on four datasets.

s	5-fold cross-validation (%)								
	9	18	27	36	45	54	63	72	81
NR	97.37	97.36	97.4	97.38	**97.42**	97.38	97.35	97.37	97.37
GPCR	94.44	**94.66**	94.44	**94.66**	94.51	**94.66**	94.59	94.59	94.59
IC	97.47	97.56	97.57	97.61	**97.63**	97.58	97.59	97.57	97.58
E	99.53	**99.54**	**99.54**	99.52	99.52	99.52	99.52	99.52	99.52
average	97.2025	97.2800	97.2375	**97.2925**	97.2700	97.2850	97.2625	97.2625	97.265

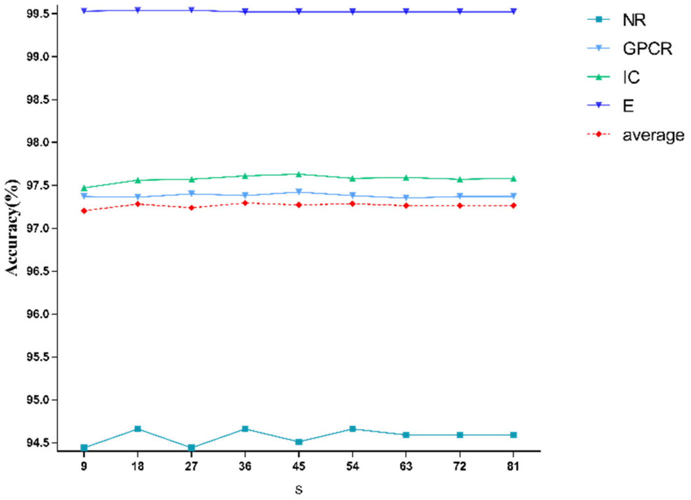

Figure S2 Prediction result of selecting different *s* on four datasets

Table S4 Prediction results on four datasets before and after Lasso for dimensionality reduction.

Dataset	LASSO	5-fold cross-validation					
		ACC(%)	SE(%)	SP(%)	F(%)	AUC	AUPR
NR	Before	94.59	24.87	99.39	36.28	0.8451	0.5162
NR	After	94.30	23.19	99.16	33.35	0.8487	0.4944
GPCR	Before	97.43	24.49	99.68	36.28	0.9296	0.4974
GPCR	After	97.43	24.33	99.68	36.13	0.9300	0.4929

IC	Before	97.62	40.26	99.67	53.80	0.9644	0.7102
IC	After	97.55	39.23	99.63	52.47	0.9656	0.7120
E	Before	99.53	54.29	99.98	69.50	0.9659	0.7984
E	After	99.52	53.70	99.98	68.95	0.9669	0.7995

Table S5 Prediction results on four datasets before and after SMOTE optimization.

Dataset	SMOTE	5-fold cross-validation					
		ACC(%)	SE(%)	SP(%)	F(%)	AUC	AUPR
NR	Before	94.30	23.19	99.16	33.35	0.8487	0.4944
NR	After	95.28	94.81	95.74	95.25	0.9886	0.9875
GPCR	Before	97.43	24.33	99.68	36.13	0.9300	0.4929
GPCR	After	96.19	95.83	96.56	96.18	0.9923	0.9923
IC	Before	97.55	39.23	99.63	52.47	0.9656	0.7120
IC	After	96.74	95.54	97.94	96.70	0.9956	0.9958
E	Before	99.52	53.70	99.98	68.95	0.9669	0.7995
E	After	98.22	97.61	98.83	98.21	0.9983	0.9984

Table S6 Prediction results on four datasets of seven classifiers.

Dataset	Method	ACC(%)	SE(%)	SP(%)	F(%)	AUC	AUPR
NR	**RF**	**95.28**	**94.81**	**95.74**	**95.25**	**0.9886**	**0.9875**
	SVM	91.20	94.07	88.33	91.43	0.9644	0.9442
	DT	89.07	89.07	89.07	89.06	0.9239	0.9043
	OR	79.81	74.26	85.37	78.41	0.7981	0.7815
	KNN	70.65	64.26	77.04	68.58	0.7822	0.7926
	RIP	86.48	88.15	84.81	86.69	0.8856	0.8695
	NB	62.69	82.22	43.15	68.81	0.7492	0.7530
GPCR	**RF**	**96.19**	**95.83**	**96.56**	**96.18**	**0.9923**	**0.9923**
	SVM	94.74	96.98	92.49	94.86	0.9867	0.9807
	DT	90.07	91.71	88.43	90.22	0.9371	0.9253
	OR	84.32	81.36	87.27	83.85	0.8432	0.8228
	KNN	92.18	96.59	87.77	92.51	0.9218	0.8797
	RIP	90.64	91.23	90.05	90.70	0.9237	0.8968
	NB	73.03	77.01	69.06	74.07	0.7970	0.7942
IC	**RF**	**96.74**	**95.54**	**97.94**	**96.70**	**0.9956**	**0.9958**
	SVM	95.04	97.50	92.57	95.16	0.9895	0.9837
	DT	90.45	90.56	90.33	90.46	0.9566	0.9585
	OR	84.71	74.76	94.66	83.01	0.8471	0.8624
	KNN	96.44	98.71	94.16	96.52	0.9645	0.9408
	RIP	92.10	92.07	92.12	92.07	0.9350	0.9290
	NB	66.50	75.94	57.06	69.39	0.7437	0.7304
E	**RF**	**98.22**	**97.61**	**98.83**	**98.21**	**0.9983**	**0.9984**
	SVM	97.15	97.81	96.50	97.17	0.9961	0.9953
	DT	93.93	94.42	93.44	93.96	0.9727	0.9715
	OR	85.78	78.66	92.90	84.69	0.8578	0.8604

**Dataset**	**Method**	**ACC(%)**	**SE(%)**	**SP(%)**	**F(%)**	**AUC**	**AUPR**
	KNN	95.93	98.89	92.97	96.05	0.9594	0.9307
	RIP	93.78	94.26	93.30	93.81	0.9502	0.9375
	NB	70.84	70.00	71.69	70.59	0.7849	0.7855

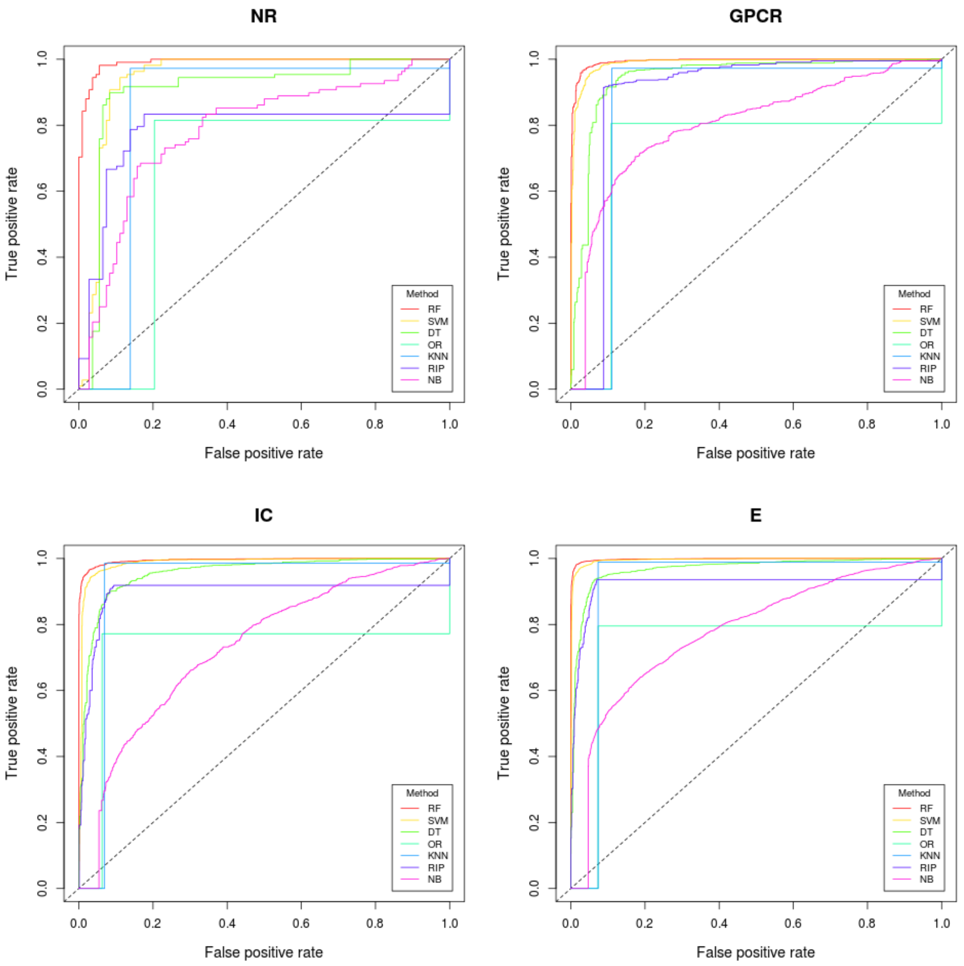

(a) Run the second 5-fold cross-validation

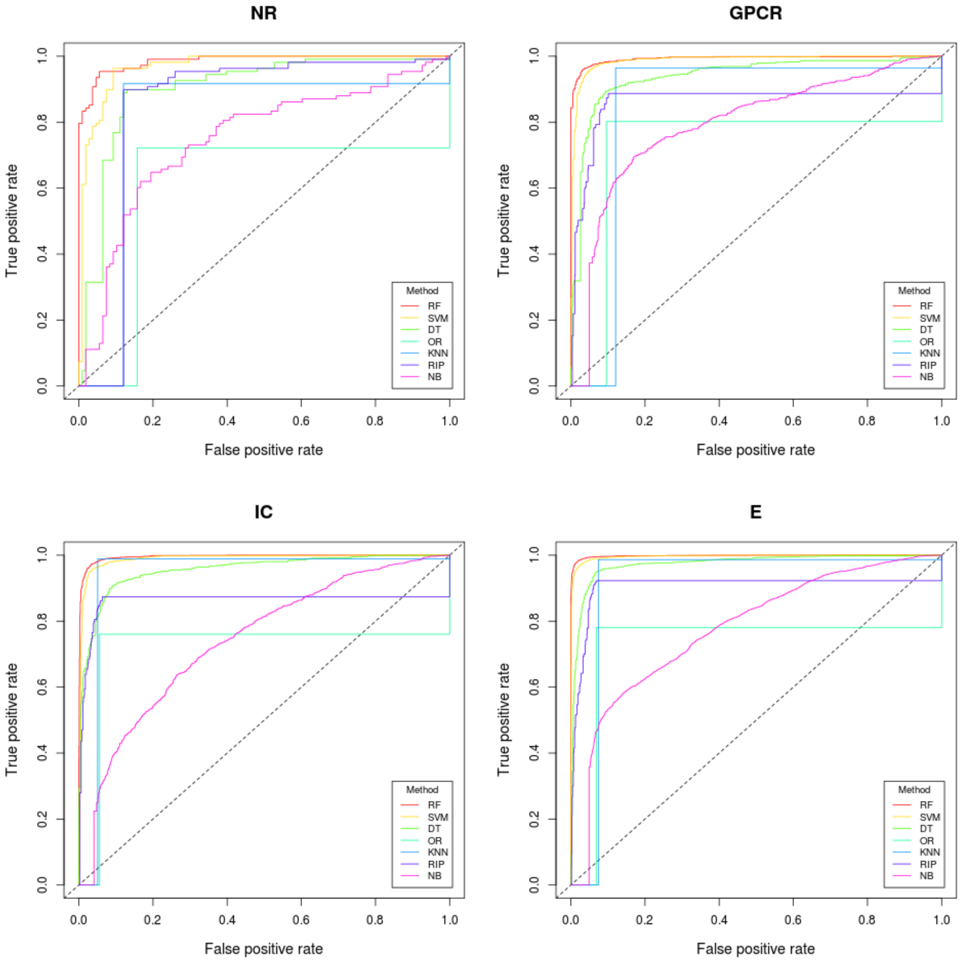

(b) Run the third 5-fold cross-validation

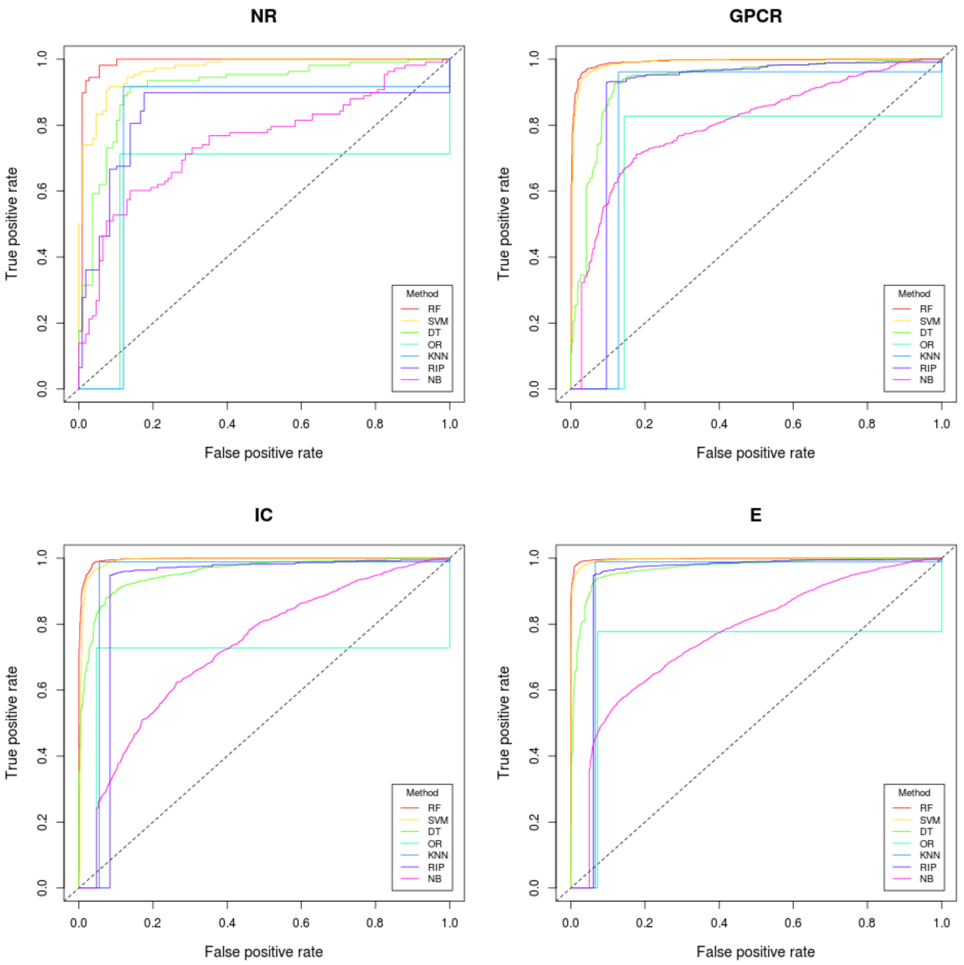

(c) Run the fourth 5-fold cross-validation

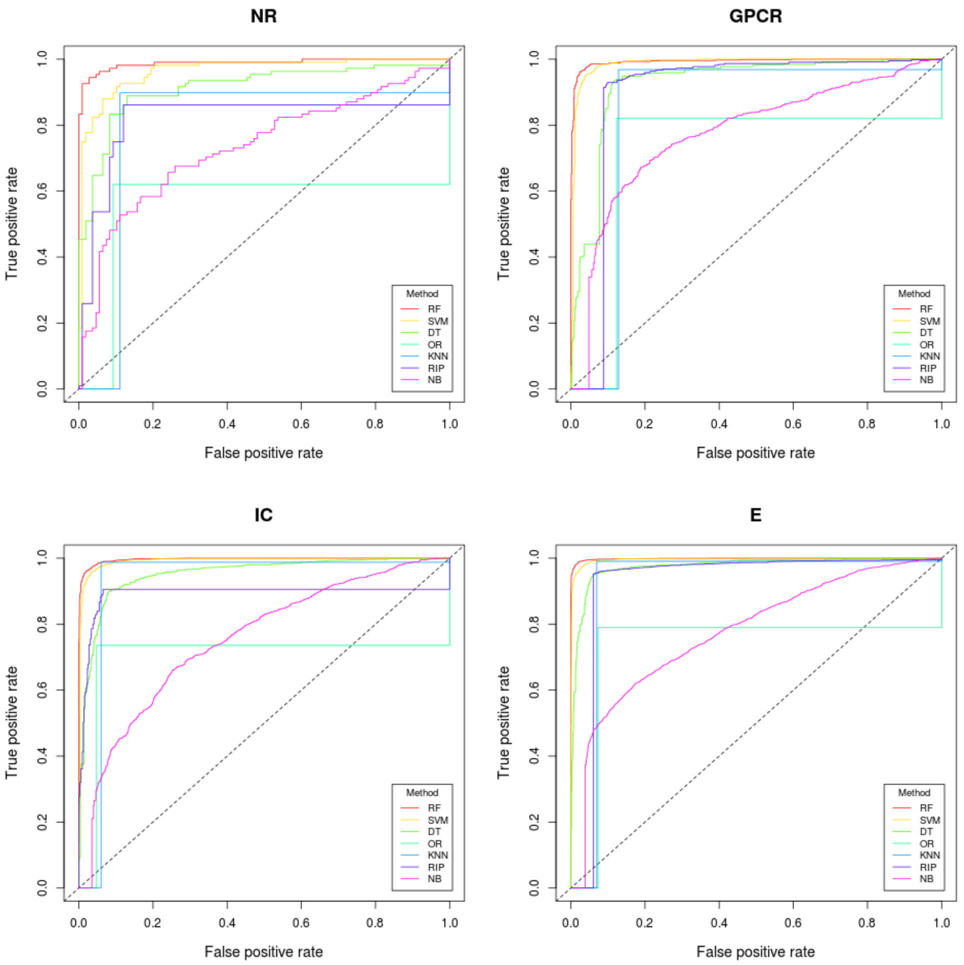

(d) Run the fifth 5-fold cross-validation

Figure S3 the ROC curves of different classifiers in 5-fold cross-validation

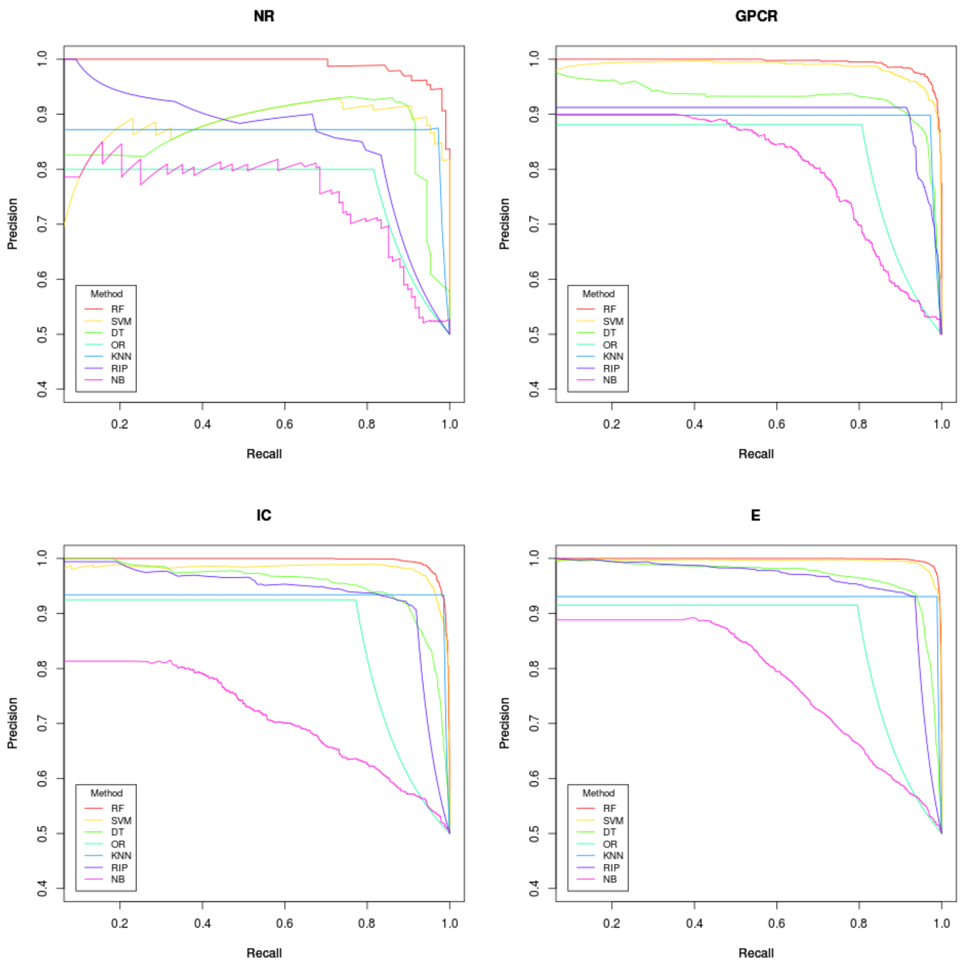

(a) Run the second 5-fold cross-validation

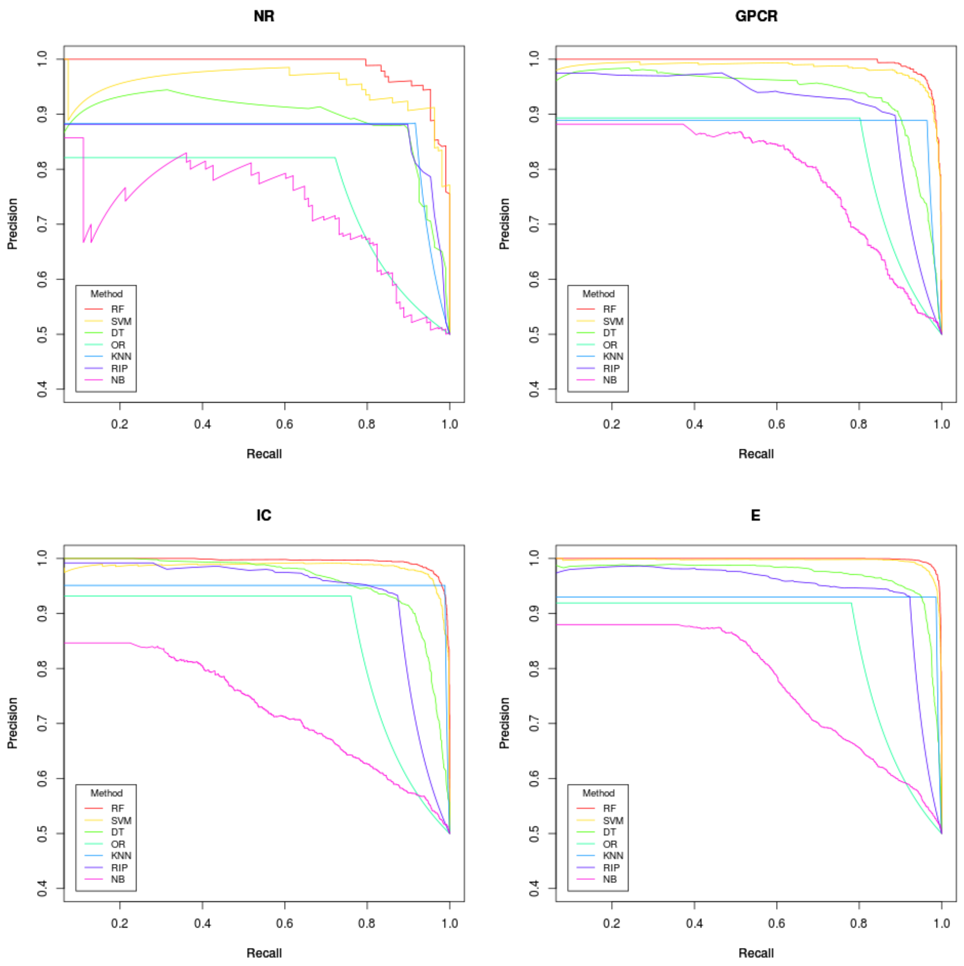

(b) Run the third 5-fold cross-validation

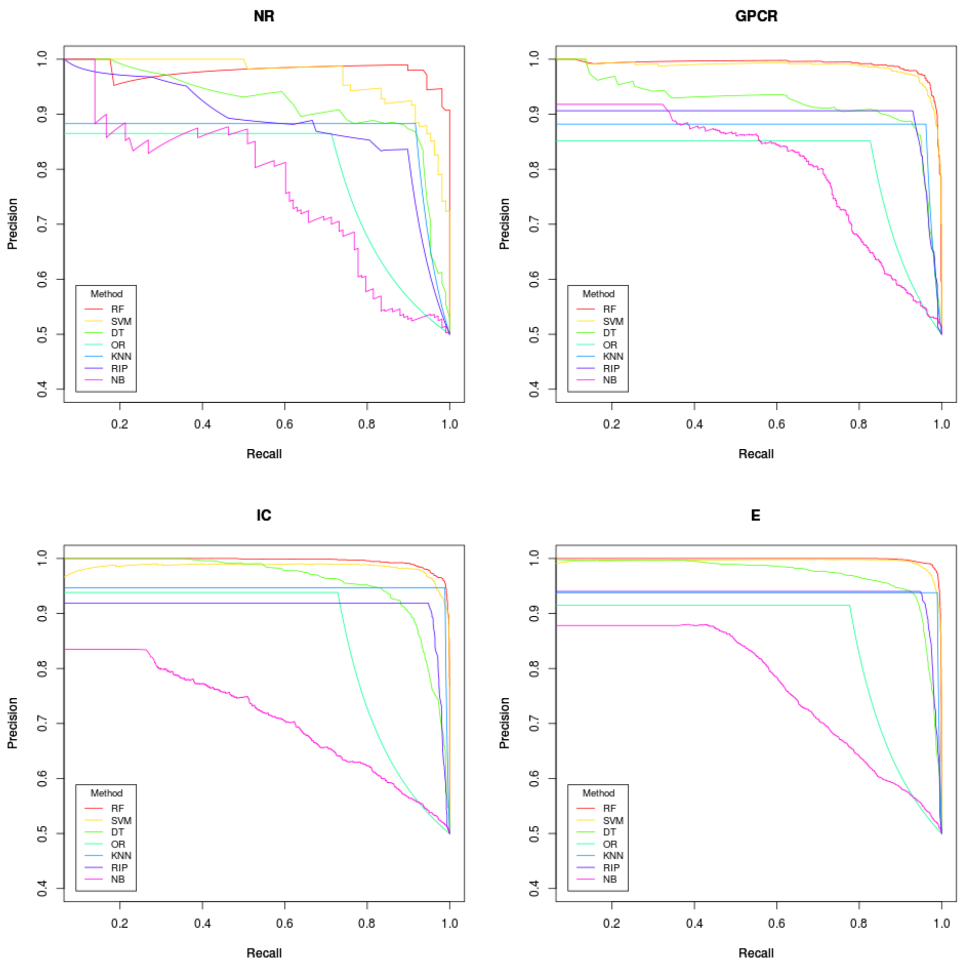

(c) Run the fourth 5-fold cross-validation

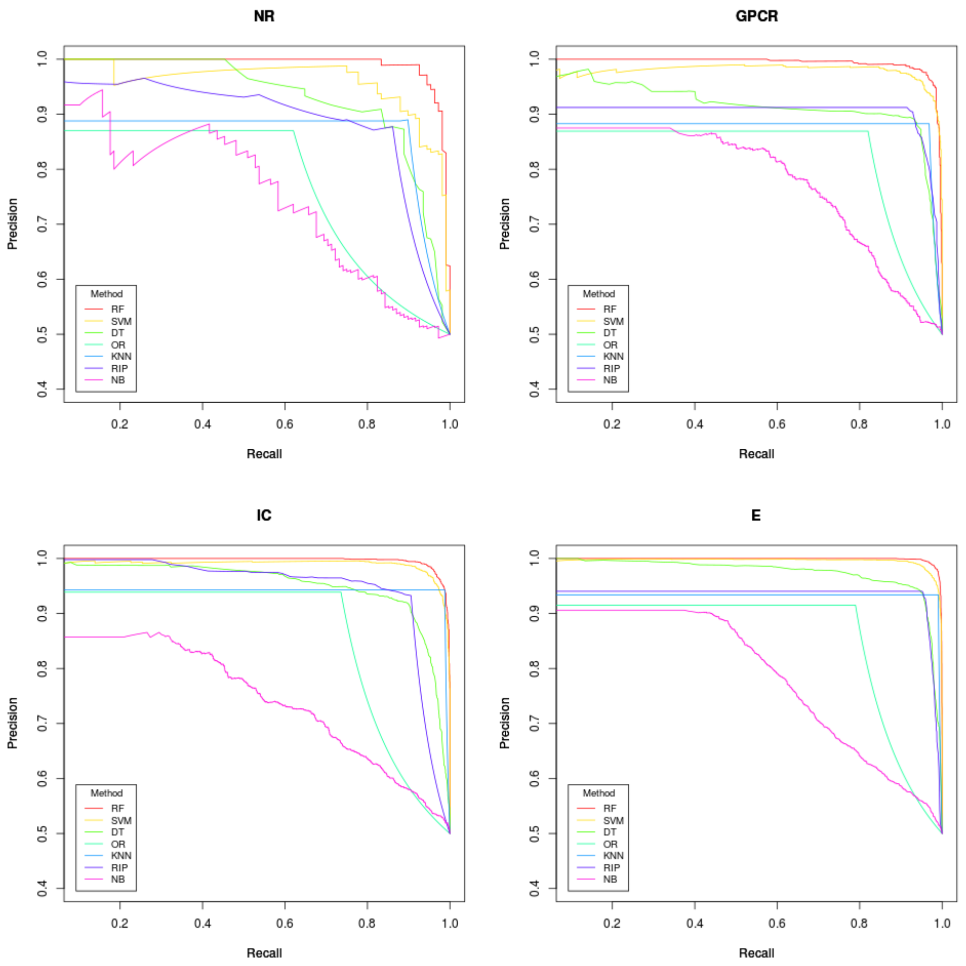

(d) Run the fifth 5-fold cross-validation

Figure S4 the PR curves of different classifiers in 5-fold cross-validation

Table S7 Drug-target interaction pairs with a probability score no less than 0.5.

Drug	Target	Prob
DB00201	hsa3783	0.988
DB00277	hsa3783	0.982
DB01412	hsa3783	0.93
DB00530	hsa238	0.886
DB00806	hsa3783	0.884
DB00824	hsa3783	0.866
DB00530	hsa2263	0.864
DB00661	hsa57719	0.846
DB01303	hsa3783	0.844
DB08916	hsa2263	0.806
DB00996	hsa56267	0.79
DB08916	hsa238	0.79
DB00864	hsa1609	0.786
DB00651	hsa3783	0.784

Drug	Target	Prob
DB00864	hsa10869	0.77
DB00091	hsa55783	0.766
DB00091	hsa10869	0.748
DB00091	hsa1609	0.744
DB00302	hsa56267	0.736
DB00799	hsa10062	0.724
DB00661	hsa219931	0.722
DB00513	hsa56267	0.718
DB01101	hsa51477	0.718
DB00230	hsa56267	0.71
DB00864	hsa3508	0.706
DB00091	hsa3508	0.704
DB00235	hsa10869	0.698
DB00740	hsa347088	0.694
DB00864	hsa4595	0.692
DB00459	hsa10062	0.692
DB00091	hsa4595	0.69
DB00864	hsa55783	0.69
DB01101	hsa4123	0.684
DB00091	hsa84909	0.674
DB00864	hsa84909	0.67
DB00091	hsa65264	0.668
DB01101	hsa1609	0.666
DB00091	hsa64409	0.664
DB00299	hsa3783	0.664
DB01100	hsa57719	0.662
DB00864	hsa64409	0.656
DB00799	hsa2063	0.656
DB00523	hsa10062	0.65
DB00755	hsa10062	0.65
DB00982	hsa10062	0.65
DB00787	hsa3783	0.65
DB00091	hsa23135	0.648
DB00864	hsa4123	0.648
DB00677	hsa1609	0.646
DB00996	hsa57719	0.64
DB00806	hsa10869	0.638
DB00864	hsa80198	0.638
DB00806	hsa51477	0.636
DB00337	hsa134510	0.634
DB00864	hsa65264	0.632
DB00230	hsa57719	0.632

Drug	Target	Prob
DB00091	hsa4123	0.63
DB00864	hsa10524	0.63
DB00548	hsa3174	0.63
DB00313	hsa3174	0.63
DB01004	hsa3783	0.628
DB00123	hsa56267	0.626
DB00806	hsa4595	0.624
DB01375	hsa3174	0.624
DB00900	hsa3783	0.624
DB00566	hsa3174	0.624
DB00996	hsa219931	0.622
DB01440	hsa3174	0.62
DB00302	hsa57719	0.62
DB00139	hsa3174	0.62
DB00235	hsa1609	0.618
DB00091	hsa10524	0.618
DB00619	hsa204851	0.614
DB00230	hsa219931	0.614
DB00337	hsa10869	0.612
DB00513	hsa57719	0.612
DB01011	hsa56898	0.61
DB00806	hsa4214	0.606
DB00864	hsa23135	0.604
DB00864	hsa204851	0.604
DB00806	hsa4123	0.602
DB05294	hsa238	0.602
DB00235	hsa4214	0.602
DB01159	hsa57719	0.6
DB00864	hsa4214	0.6
DB00806	hsa10678	0.598
DB00091	hsa56852	0.598
DB00337	hsa1609	0.596
DB00619	hsa10869	0.596
DB00740	hsa165829	0.596
DB00864	hsa55768	0.596
DB00326	hsa219931	0.596
DB00235	hsa4595	0.594
DB00864	hsa56852	0.594
DB00619	hsa4214	0.594
DB00091	hsa204851	0.594
DB00677	hsa1521	0.592
DB00568	hsa57719	0.59

Drug	Target	Prob
DB00201	hsa51477	0.59
DB00235	hsa10524	0.588
DB00302	hsa219931	0.586
DB00677	hsa4123	0.584
DB00677	hsa10893	0.584
DB00577	hsa3783	0.582
DB00091	hsa4214	0.582
DB00235	hsa10678	0.58
DB00619	hsa84909	0.58
DB01101	hsa55783	0.58
DB00091	hsa55768	0.58
DB00806	hsa1609	0.578
DB04841	hsa57719	0.578
DB00548	hsa57719	0.578
DB00513	hsa219931	0.578
DB00255	hsa2063	0.578
DB00235	hsa56852	0.576
DB00235	hsa10893	0.574
DB00864	hsa2242	0.574
DB06777	hsa219931	0.574
DB06777	hsa57719	0.574
DB01586	hsa57719	0.574
DB01586	hsa219931	0.574
DB00858	hsa57719	0.574
DB01159	hsa219931	0.572
DB00201	hsa10869	0.57
DB00457	hsa238	0.57
DB01101	hsa4214	0.57
DB00858	hsa219931	0.57
DB00590	hsa238	0.568
DB01412	hsa51477	0.568
DB01100	hsa219931	0.568
DB00806	hsa80198	0.568
DB00632	hsa3174	0.568
DB00548	hsa219931	0.568
DB00513	hsa4123	0.566
DB00123	hsa219931	0.566
DB00619	hsa841	0.564
DB01115	hsa57719	0.564
DB00619	hsa64409	0.562
DB00677	hsa10678	0.562
DB00677	hsa10869	0.562

Drug	Target	Prob
DB05294	hsa2263	0.562
DB01482	hsa3783	0.562
DB00568	hsa219931	0.562
DB00513	hsa25902	0.56
DB01610	hsa3783	0.56
DB00091	hsa80198	0.56
DB00742	hsa219931	0.558
DB00174	hsa3174	0.558
DB00091	hsa399818	0.556
DB00544	hsa25902	0.556
DB00280	hsa57719	0.556
DB00235	hsa51477	0.556
DB00128	hsa3174	0.556
DB00337	hsa4595	0.554
DB04868	hsa2242	0.554
DB01612	hsa3174	0.554
DB01184	hsa57719	0.554
DB00619	hsa3508	0.554
DB05389	hsa3174	0.552
DB01375	hsa57719	0.552
DB01375	hsa219931	0.552
DB00825	hsa219931	0.552
DB00337	hsa3508	0.552
DB00201	hsa4214	0.552
DB00864	hsa841	0.55
DB01101	hsa84909	0.55
DB04841	hsa219931	0.55
DB00806	hsa10524	0.55
DB01101	hsa4595	0.548
DB00162	hsa10062	0.548
DB00255	hsa10062	0.548
DB00235	hsa80198	0.548
DB00166	hsa219931	0.548
DB00855	hsa56267	0.546
DB00632	hsa219931	0.546
DB00201	hsa116535	0.546
DB00166	hsa57719	0.546
DB00230	hsa25902	0.544
DB00401	hsa57719	0.544
DB00167	hsa3174	0.544
DB00161	hsa3174	0.544
DB00149	hsa3174	0.544

Drug	Target	Prob
DB00123	hsa57719	0.544
DB00230	hsa4123	0.542
DB01101	hsa81490	0.542
DB01412	hsa10869	0.542
DB00740	hsa1394	0.542
DB00859	hsa3174	0.542
DB00472	hsa57719	0.542
DB00166	hsa3174	0.542
DB03166	hsa3174	0.54
DB00258	hsa3174	0.54
DB00235	hsa55768	0.54
DB00201	hsa10678	0.538
DB00235	hsa84909	0.538
DB00619	hsa4595	0.538
DB00864	hsa238	0.538
DB01101	hsa399818	0.538
DB00884	hsa10062	0.538
DB01412	hsa116535	0.538
DB00513	hsa54858	0.538
DB00230	hsa3174	0.538
DB00806	hsa10893	0.536
DB00996	hsa25902	0.536
DB06782	hsa3174	0.536
DB00855	hsa3174	0.536
DB00855	hsa57719	0.536
DB00401	hsa219931	0.536
DB00251	hsa3783	0.536
DB00149	hsa56267	0.534
DB00167	hsa56267	0.534
DB01412	hsa4214	0.534
DB01054	hsa219931	0.534
DB00677	hsa65264	0.534
DB00235	hsa65264	0.534
DB00235	hsa4123	0.532
DB01412	hsa10678	0.532
DB06768	hsa3174	0.532
DB04938	hsa2063	0.532
DB01440	hsa219931	0.532
DB01101	hsa65264	0.532
DB00985	hsa3783	0.532
DB00909	hsa219931	0.532
DB00898	hsa3174	0.532

Drug	Target	Prob
DB00513	hsa3174	0.532
DB00313	hsa219931	0.532
DB00130	hsa56267	0.53
DB00337	hsa84909	0.53
DB00619	hsa1609	0.53
DB01101	hsa64409	0.53
DB00651	hsa4214	0.53
DB00139	hsa219931	0.53
DB00302	hsa4123	0.528
DB04868	hsa238	0.528
DB01233	hsa116535	0.528
DB01115	hsa219931	0.528
DB01101	hsa55768	0.528
DB00677	hsa54858	0.528
DB00622	hsa219931	0.528
DB00091	hsa27068	0.528
DB00129	hsa56267	0.526
DB00855	hsa4123	0.526
DB00313	hsa57719	0.526
DB00130	hsa4123	0.524
DB08944	hsa57719	0.524
DB01159	hsa3174	0.524
DB01101	hsa26227	0.524
DB00971	hsa3174	0.524
DB00529	hsa3174	0.524
DB00393	hsa219931	0.524
DB00996	hsa4123	0.522
DB01189	hsa3174	0.522
DB01101	hsa5226	0.522
DB00855	hsa219931	0.522
DB00735	hsa57719	0.522
DB00442	hsa3783	0.522
DB00228	hsa3174	0.522
DB00201	hsa1609	0.52
DB00201	hsa4595	0.52
DB00235	hsa23135	0.52
DB01101	hsa10678	0.52
DB00825	hsa57719	0.52
DB00806	hsa65264	0.52
DB00653	hsa3174	0.52
DB00566	hsa219931	0.52
DB00337	hsa10524	0.52

Drug	Target	Prob
DB00277	hsa51477	0.52
DB00129	hsa3174	0.52
DB00129	hsa4123	0.518
DB00235	hsa64409	0.518
DB00302	hsa25902	0.518
DB01620	hsa57719	0.518
DB00835	hsa57719	0.518
DB00806	hsa55768	0.518
DB00459	hsa2063	0.518
DB00405	hsa57719	0.518
DB00130	hsa219931	0.518
DB00123	hsa3174	0.518
DB00201	hsa10893	0.516
DB00513	hsa1609	0.516
DB01101	hsa10869	0.516
DB01412	hsa10893	0.516
DB08913	hsa3174	0.516
DB08894	hsa3174	0.516
DB06723	hsa3174	0.516
DB06439	hsa3174	0.516
DB01593	hsa3174	0.516
DB01592	hsa3174	0.516
DB01390	hsa3174	0.516
DB01377	hsa3174	0.516
DB01370	hsa3174	0.516
DB01356	hsa3174	0.516
DB01344	hsa3174	0.516
DB01303	hsa51477	0.516
DB01223	hsa51477	0.516
DB01164	hsa3174	0.516
DB01109	hsa3174	0.516
DB01093	hsa3174	0.516
DB01049	hsa3174	0.516
DB00994	hsa3174	0.516
DB00930	hsa3174	0.516
DB00761	hsa3174	0.516
DB00707	hsa3174	0.516
DB00619	hsa80198	0.516
DB00528	hsa219931	0.516
DB00516	hsa3174	0.516
DB00435	hsa3174	0.516
DB00407	hsa3174	0.516

Drug	Target	Prob
DB00375	hsa3174	0.516
DB00286	hsa3174	0.516
DB00123	hsa4123	0.514
DB00946	hsa2063	0.514
DB00632	hsa57719	0.514
DB00128	hsa219931	0.514
DB00337	hsa23135	0.512
DB00502	hsa56898	0.512
DB00619	hsa89122	0.512
DB00677	hsa84909	0.512
DB00746	hsa10893	0.512
DB00334	hsa165829	0.512
DB05389	hsa219931	0.512
DB01169	hsa3174	0.512
DB00815	hsa3174	0.512
DB00669	hsa116535	0.512
DB00515	hsa3174	0.512
DB00464	hsa3174	0.512
DB00343	hsa219931	0.512
DB00230	hsa54858	0.512
DB00129	hsa219931	0.512
DB00677	hsa64409	0.51
DB00746	hsa10678	0.51
DB01224	hsa10062	0.51
DB00864	hsa5226	0.51
DB00621	hsa219931	0.51
DB00270	hsa219931	0.51
DB00174	hsa56267	0.508
DB01195	hsa219931	0.508
DB01167	hsa3783	0.508
DB01101	hsa54858	0.508
DB01031	hsa57719	0.508
DB00996	hsa5226	0.508
DB00235	hsa100913187	0.506
DB00277	hsa10869	0.506
DB00806	hsa64409	0.506
DB01440	hsa57719	0.506
DB01054	hsa57719	0.506
DB00651	hsa51477	0.506
DB00326	hsa57719	0.506
DB00277	hsa4214	0.506
DB00266	hsa2063	0.506

Drug	Target	Prob
DB00167	hsa219931	0.506
DB00149	hsa219931	0.506
DB00119	hsa3174	0.506
DB00824	hsa4214	0.504
DB00824	hsa51477	0.504
DB00756	hsa2063	0.504
DB00556	hsa3174	0.504
DB00258	hsa219931	0.504
DB00201	hsa10524	0.504
DB00174	hsa219931	0.504
DB00091	hsa5226	0.504
DB00123	hsa25902	0.502
DB00213	hsa10062	0.502
DB04938	hsa10062	0.502
DB01113	hsa57719	0.502
DB01080	hsa57719	0.502
DB01080	hsa219931	0.502
DB01028	hsa3174	0.502
DB00855	hsa5226	0.502
DB00740	hsa219931	0.502
DB00566	hsa57719	0.502
DB00457	hsa2263	0.502
DB00337	hsa4214	0.502
DB00257	hsa57719	0.502
DB00172	hsa3174	0.502
DB00167	hsa5226	0.502
DB00149	hsa5226	0.502
DB00145	hsa219931	0.502
DB00133	hsa219931	0.502
DB01114	hsa57719	0.5
DB00918	hsa116535	0.5
DB00806	hsa56852	0.5
DB00091	hsa54858	0.5

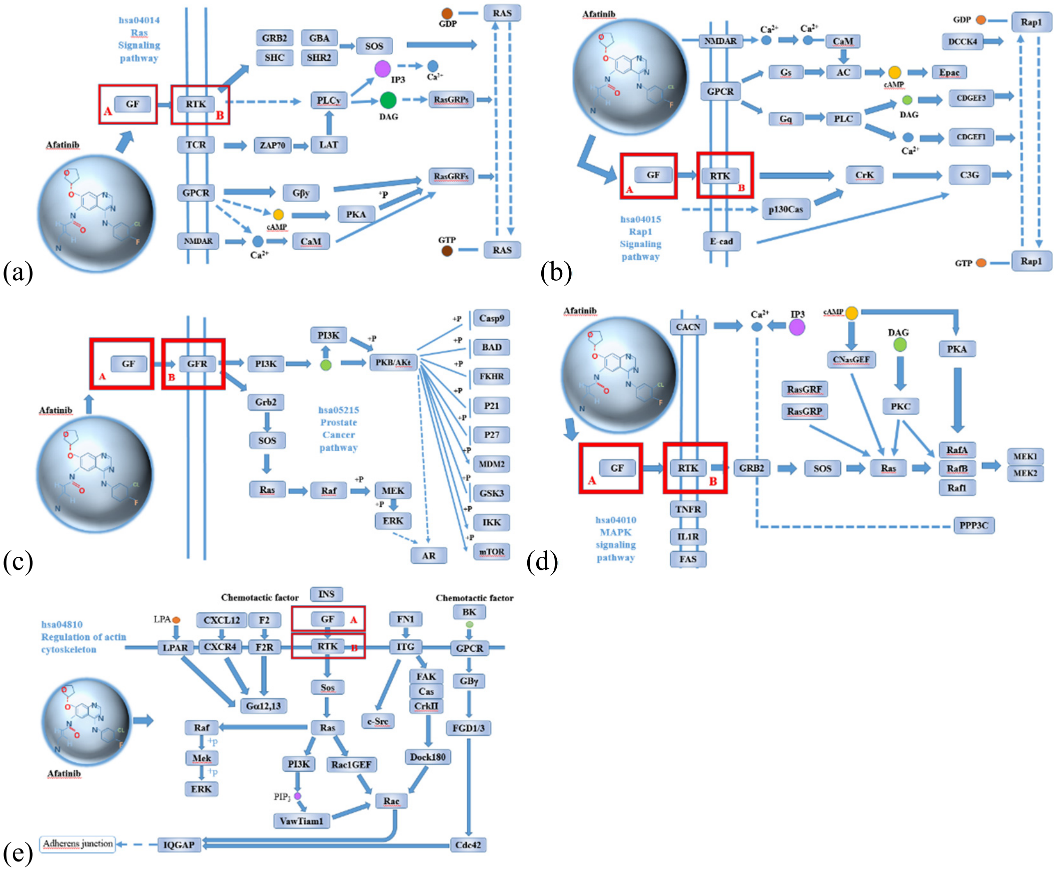

Figure S5 the inferred evidence for DTIs of afatinib (DB08916) and FGFR2 (hsa2263). In the Figure S5(a)RTK contains EGFR, FGFR2, GF contains EGF; in the Figure S5(b)GF contains EGF, RTK contains FGFR2, EGFR; in the Figure S5(c)GF contains EGF, GFR contains EGFR, ERBB2, FGFR2; in the Figure S5(d) RTK contains EGFR, ERBB2, ERBB4, FGFR2, GF contains EGF ; in the Figure S5(e), RTK contains FGFR2, EGFR,GF contains EGF
